# Photodegradation of Turmeric Oleoresin Under Fluorescent Light and White LED: Impacts on the Chemical Stability, Bioactivity, and Photosensitizing Property of Curcuminoids

**DOI:** 10.3390/molecules30153187

**Published:** 2025-07-30

**Authors:** Heejeong Kim, Juyeon Oh, Jungil Hong

**Affiliations:** Department of Food Science and Technology, College of Science and Convergence Technology, Seoul Women’s University, 621 Hwarangro, Nowon-gu, Seoul 01797, Republic of Korea; heej9812@swu.ac.kr (H.K.); ojo551945@swu.ac.kr (J.O.)

**Keywords:** turmeric oleoresin, curcuminoid, curcumin, photodegradation, photosensitivity, white LED, fluorescent light

## Abstract

Turmeric oleoresin (TO), a natural pigment derived from *Curcuma longa* rhizomes, is valued for its health benefits, which are primarily attributed to its rich curcuminoid content (curcumin, demethoxycurcumin, and bisdemethoxycurcumin). Despite these benefits, curcuminoids are known to be light-sensitive and possess photosensitizing properties. This study investigated the impact of common light sources, fluorescent light and white LED (both at 10 W/m^2^), on the chemical stability, antioxidant activity, cytotoxicity, and photosensitizing properties of TO. Exposure to both light sources significantly reduced TO’s color and fluorescence intensity, with white LED causing greater instability. HPLC analysis confirmed a decrease in individual curcuminoid levels, with curcumin degrading most rapidly under both conditions. The DPPH radical scavenging activity of irradiated TO decreased compared to fresh or dark-stored turmeric, whereas its ABTS radical scavenging activity increased upon light exposure. Photosensitizing potency, measured by formazan decolorization and lipid peroxide formation, declined as TO decomposed under light. Conversely, the cytotoxicity of TO against B16F10 melanoma cells was significantly enhanced under light exposure, though this effect was diminished significantly after 24 h of pre-irradiation. These findings underscore the instability of turmeric pigment under common lighting conditions, which should be a crucial consideration when processing, storing, and distributing turmeric-containing products.

## 1. Introduction

**Figure 1:**
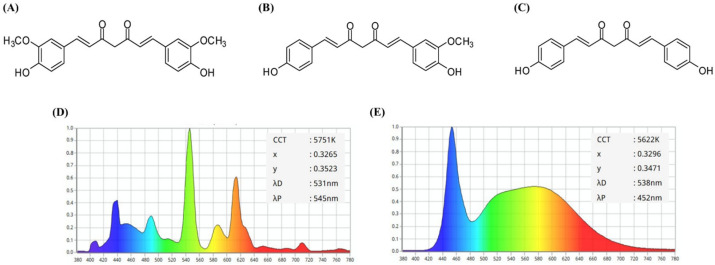
Structures of curcuminoids in turmeric oleoresin, including (**A**) curcumin, (**B**) DMC, and (**C**) BMC, and emission spectrum profiles of fluorescent light (**D**) and white LED (**E**) used in the present study.

Turmeric (*Curcuma longa* Linné) is widely used as a food additive to enhance flavor, taste, and preservation [1]. The primary active components contributing to its vibrant yellow color and biological activity are curcuminoids including curcumin, demethoxycurcumin (DMC), and bisdemethoxycurcumin (BMC) (Figure 1A–C) [2]. Among these, curcumin, the most abundant curcuminoid, possesses potent antioxidant properties primarily due to its phenolic moiety, which is capable of donating H-atoms and protecting cell membranes from oxidative stress [3,4]. Curcumin exhibits a variety of biological activities, including anti-inflammatory, anticancer, and immunomodulatory effects [3,4,5,6]. DMC and BMC have also been reported to possess various health benefits [2,3,7].

Turmeric oleoresin (TO), an organic solvent extracted from dried *C. longa* rhizome, is particularly rich in curcuminoids [8]. Consequently, TO finds widespread application across the food, pharmaceutical, and cosmetics industries [9,10]. Despite their beneficial properties, curcuminoids are chemically unstable, with their stability being significantly influenced by environmental factors such as pH, temperature, and light exposure [11,12,13]. Previous studies have reported that the chemical stability of curcuminoids rapidly degrades under alkaline conditions and elevated temperatures [14]. Notably, exposure to light including UV, sunlight, or fluorescent light has been reported to accelerate the photodegradation of curcuminoids, yielding smaller phenolic products such as vanillin and ferulic acid [15,16,17]. Furthermore, the kinetics and profiles of curcumin photodegradation are strongly affected by the surrounding medium and specific wavelengths of light [18,19].

Light-emitting diodes (LEDs), recognized for their energy efficiency and minimal heat generation, emit across a broad visible spectrum (400–760 nm) and are widely employed in both consumer and industrial environments [20]. Among these, white LEDs are particularly prevalent due to their broad spectral emission—including red, green, and blue light—and lower cost compared to monochromatic LEDs [21]. Given the continuous exposure of food products to diverse lighting systems throughout their processing, storage, and distribution stages, understanding the impact of such irradiation on food constituents is of increasing importance.

Maintaining the stability and integrity of food pigments is crucial for preserving product appeal and quality. However, many dietary pigments undergo photodegradation upon interaction with light, leading to undesirable changes in the properties of food ingredients. Photoreactive compounds such as riboflavin, chlorophyll, and porphyrins can, for instance, induce lipid oxidation upon light exposure [22]. Similarly, curcuminoids have the potential to undergo alterations in their chemical and functional properties when interacting with different types of light [16,17,23]. The light-induced decomposition of curcuminoids is well established [23]. However, limited research has explored the effects of white LED and fluorescent light exposure, the most commonly applied light sources, on the chemical, biological, and photodynamic properties of turmeric pigment. Furthermore, TO has been reported to induce phototoxicity in melanoma cells [24]; the effect of TO photodegradation on this phenomenon remains unreported. Therefore, the present study evaluated the changes in chemical stability, antioxidant activity, photosensitizing property, and cell phototoxicity of TO under fluorescent light and white LED irradiation, as these are frequently encountered and commonly applied light sources during food storage and distribution.

## 2. Results and Discussion

### 2.1. Changes in the Photostability of TO Under Light Irradiation

Many natural plant pigments are known to be unstable under light. Chlorophylls decompose via generating singlet oxygen under light, whereas carotenoids undergo oxidation and isomerization [25,26]. This study initially evaluated the photostability of turmeric pigment in TO under two commonly applied light sources, a fluorescent light and white LED. The emission wavelength spectra of the fluorescent light and white LED used in this study are shown in Figure 1D and Figure 1E, respectively. To confirm photostability, changes in absorption and fluorescence properties of TO were assessed. Changes in color intensity of different concentrations (20–1000 μg/mL) of TO dissolved in dimethyl sulfoxide (DMSO) were analyzed under 10 W/m^2^ fluorescent light or white LED at 25 °C for 24 h. Based on a previous study showing a peak absorption wavelength of TO at 435 nm in DMSO, its photostability was evaluated by measuring absorbance changes at 435 nm [16].

**Figure 2:**
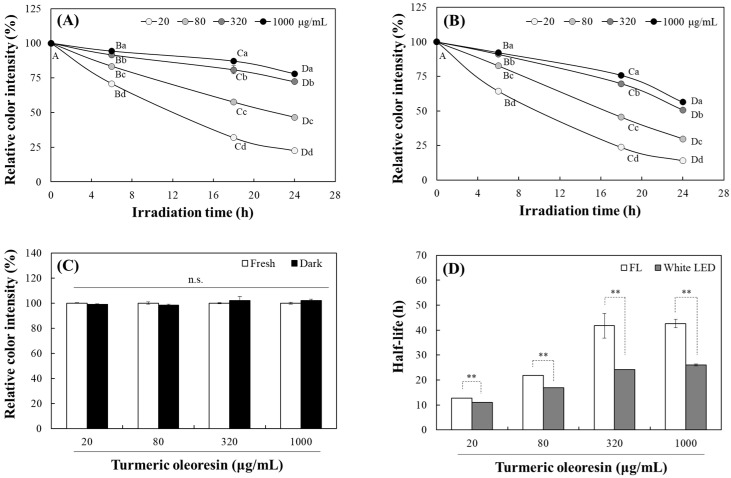
Color stability of TO under irradiation with fluorescent light or white LED. Changes in the color intensity of turmeric pigment under fluorescent light (FL) (**A**), white LED (**B**), or in the dark (**C**) over 24 h were measured at 435 nm. Half-lives (time required for 50% of color reduction) were also calculated (**D**). Each value represents the mean ± SD (*n* = 3). Different uppercase and lowercase letters indicate significant difference among different irradiation times and different concentrations, respectively (*p* < 0.05), based on a one-way ANOVA with Tukey’s HSD test. n.s.—not significant. **—significantly different between fluorescent light and white LED according to Student’s *t*-test (** *p* < 0.01).

The color intensity of TO gradually decreased with increasing irradiation time under both fluorescent light and white LED. After 24 h irradiation, the residual color intensities at 20, 80, 320, and 1000 μg/mL of TO compared to initial levels were 22.4, 46.5, 72.3, and 78.0% under fluorescent light (Figure 2A) and 14.1, 29.8, 50.7, and 56.4% under white LED (Figure 2B), respectively. The photostability of TO increased significantly with higher concentrations. However, no significant color degradation was observed after 24 h incubation at all TO concentrations in the dark, indicating that turmeric pigment is relatively stable in organic solvents in the absence of light (Figure 2C). The half-life, which is the irradiation time period required for 50% color reduction, was significantly shorter under white LED than under fluorescent light at all TO concentrations (Figure 2D); this indicates that TO is more sensitive to white LED than fluorescent light.

The yellow color of TO is attributed to its symmetric α, β-unsaturated carbonyl structure [27]. Exposure to light induces photooxidation of this structure, resulting in color reduction of TO. Since higher TO concentrations resulted in higher photostability in this experiment, this delay in pigment decomposition at high concentrations may be due to strengthened intra- or intermolecular bonding and increased light resistance via a screening effect.

**Figure 3:**
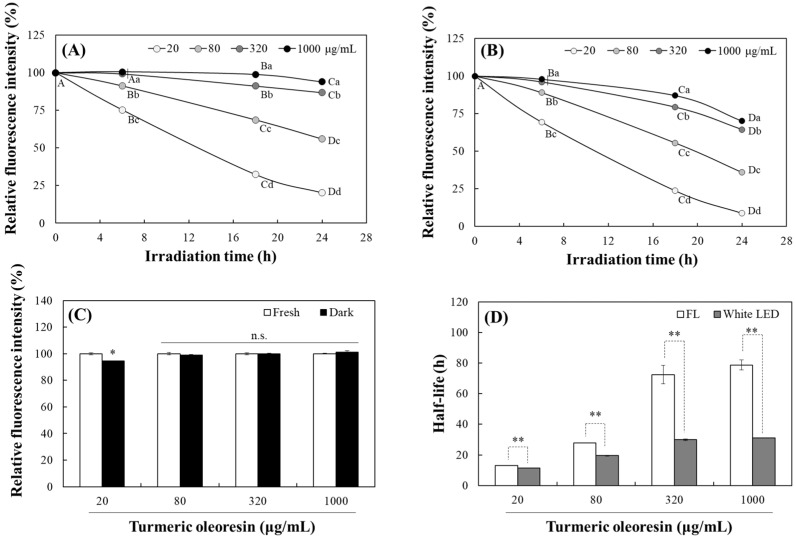
Changes in fluorescence intensity of TO under irradiation with fluorescent light (FL) or white LED. Changes in fluorescence intensity of turmeric pigment under fluorescent light (**A**), white LED (**B**), or in the dark (**C**) over 24 h were measured at an excitation of 440 nm and emission of 535 nm. Half-lives were also calculated (**D**). Each value represents the mean ± SD (*n* = 3). Different uppercase and lowercase letters indicate significant difference among different irradiation times and different concentrations, respectively (*p* < 0.05), based on a one-way ANOVA with Tukey’s HSD test. n.s.—not significant. *, **—significantly different between fresh and dark or fluorescent light and white LED according to Student’s *t*-test (* *p* < 0.05, ** *p* < 0.01).

After absorbing light, electrons in curcuminoid molecules transition to an excited state. When they return to the ground state, specific fluorescence is emitted [28]. The fluorescence characteristics of curcuminoids depend on their structural state in solvents. Accordingly, the photostability of TO dissolved in DMSO was also evaluated by analyzing its fluorescence at an excitation and emission wavelength of 440 and 535 nm, respectively (cut-off 530 nm). The degradation of TO fluorescence intensity under both light sources followed a pattern similar to that of the TO absorbance. The fluorescence intensity of TO also decreased gradually with increasing exposure time under fluorescent light (Figure 3A) or white LED (Figure 3B), showing a significant delay at higher concentrations. After 24 h irradiation, the residual fluorescence intensity at 20, 80, 320, and 1000 μg/mL of TO was 20.2, 55.9, 86.8, and 93.9% under fluorescent light and 8.70, 36.0, 64.4, and 70.1% under the white LED, respectively. No significant difference was observed at any concentrations after 24 h in the dark, except for a 5.5% decrease at 20 μg/mL (Figure 3C). The half-lives for fluorescence degradation also showed a similar pattern to the absorbance results, with the TO photodegradation being more pronounced under the white LED at all concentrations (Figure 3D).

Overall, exposure to both fluorescent light and white LED induced a significant decline in the absorbance and fluorescence of TO. The greater reduction observed under white LED exposure indicates that TO is more susceptible to this light source. This sensitivity likely arises from the stronger emission of blue light from the white LED (Figure 1D,E), which has a higher energy level and more effectively drives the photochemical degradation of curcuminoid chromophores. These results have important implications for the use of turmeric pigment in food and related products. Higher concentrations of the TO preparation may be more efficient in counteracting light-induced loss of color or fluorescence.

### 2.2. Analysis of the Effects on Individual Curcuminoid Levels During Light Irradiation

**Figure 4:**
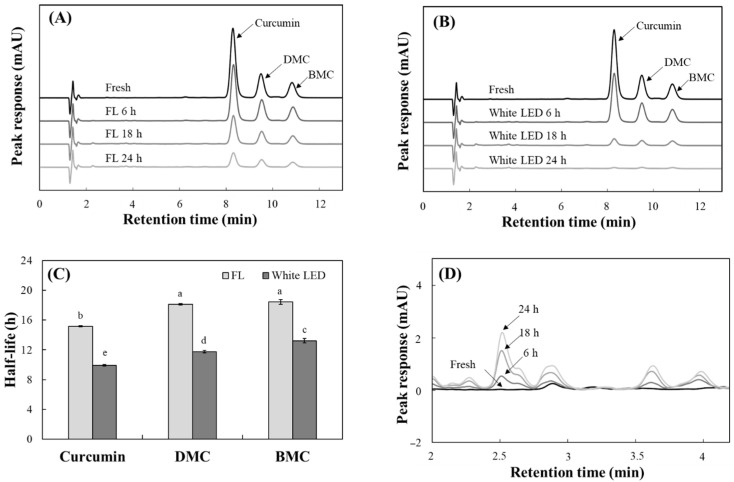
Changes of individual curcuminoid levels in turmeric pigment by light irradiation. Chromatograms of each curcuminoid in TO (20 μg/mL) and their changes by irradiation of fluorescent light (FL) (**A**) or white LED (**B**) over 24 h were analyzed using the current HPLC. Half-lives were also calculated (**C**). Formation of a photodegradation product from TO under white LED irradiation was also detected at 300 nm (**D**). Each value represents the mean ± SD (*n* = 3). Different letters indicate a significant difference (*p* < 0.05) based on a one-way ANOVA with Tukey’s HSD test.

Curcuminoids in TO have different chemical structures and contents. They are classified into three major compounds including curcumin with two methoxyl groups, DMC with one, and BMC without methoxyl group. Commercially available TO generally contains the most curcumin. Accordingly, changes in the level of individual curcuminoids in TO under both light irradiations were also evaluated using HPLC. The TO used in this study contained 40.3% curcuminoids (*w*/*w*), HPLC analysis detected three curcuminoids (curcumin, DMC, and BMC) with retention times of 8.27, 9.47, and 10.8 min and contents of 61.3, 22.6, and 16.1%, respectively (Figure 4).

The levels of curcumin, DMC and BMC in TO decreased progressively under the both fluorescent light (Figure 4A) and white LED (Figure 4B) over 24 h of irradiation. The half-lives of these curcuminoids under fluorescent light were calculated to be 15.1 for curcumin, 18.1 for DMC, and 18.4 h for BMC. Under white LED irradiation, however, these periods decreased to 9.9, 11.7 and 13.2 h, respectively (Figure 4C). In all cases, photodegradation of three curcuminoids occurred more quickly under white LED than under fluorescent light, suggesting that curcuminoids are less stable under white LED light. Of the curcuminoids, curcumin had the shortest half-life when exposed to both types of irradiations, indicating that it is the most susceptible to light.

Concurrent with the loss of parent curcuminoids, new peaks emerged at ~4.0 min of the chromatogram, which were detected in the 300 nm UV colorless region, and their intensity increased as the irradiation time was extended (Figure 4D). Previous studies have shown that curcumin photodegradation yields smaller phenolic compounds, including vanillin, ferulic aldehyde, ferulic acid, and vanillic acid [29,30,31]. These compounds are more hydrophilic and have a lower molecular weight than their parent compounds, resulting in earlier elution in reversed-phase HPLC systems. Therefore, the presence of these peaks at earlier retention times is consistent with the formation of these degradation products.

Similar to the changes in absorbance and fluorescence in TO (Figure 2 and Figure 3), the decomposition of the three curcuminoids analyzed by HPLC was more pronounced under the white LED with increased irradiation time. A previous study reported that the β-diketone moiety is primarily responsible for curcumin photodegradation [32]. Additionally, the presence of a methoxy group on the phenolic ring accelerates curcuminoid photodegradation [30]. The current results also show that, among curcuminoids sharing the common β-diketone moiety, BMC without a methoxy group was the most stable under both lights with the longest half-life. Therefore, the photostability of TO is strongly influenced by curcumin, its most abundant component. TO containing a lower curcumin level is expected to be more stable under light irradiation.

### 2.3. Changes in Antioxidant Activity of TO by Photodegradation

**Figure 5:**
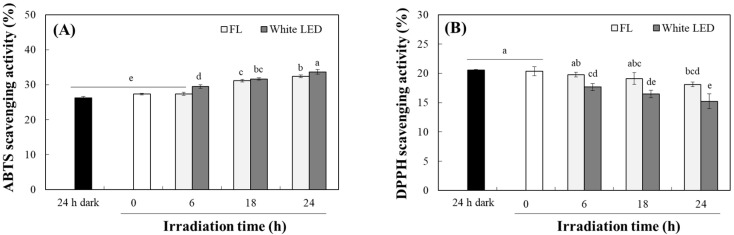
Changes in antioxidant activities of TO under light irradiation. Scavenging activities were analyzed against ABTS (**A**) and DPPH (**B**) radicals of TO (10 μg/mL in A or 20 μg/mL in (**B**) and its photodegradation products under fluorescent light (FL) and white LED (10 W/m^2^) for 6, 18, and 24 h or in the dark for 24 h. Each value represents the mean ± SD (*n* = 3). Different letters indicate a significant difference (*p* < 0.05) based on a one-way ANOVA with Tukey’s HSD test.

Curcuminoids, the principal phenolic pigments in TO, are well known for their antioxidant activity. To determine the effect of photodegradation on this activity, 2,2′-azino-bis-(3-ethyl benzothiazoline-6-sulfonic acid) (ABTS) and 2,2-diphenyl-2-picrylhydrazyl (DPPH) radical scavenging assays were performed on TO after being irradiated with fluorescent light or a white LED for 24 h. The scavenging activity of TO against the ABTS radical did not change after 24 h incubation in the dark; it increased progressively with increasing exposure time to light (Figure 5A). In contrast, the DPPH radical scavenging activity of TO decreased significantly over time with exposure to both lights. Exposure to fluorescent light reduced DPPH radical scavenging activity by 2.86, 6.08 and 11.1% after 6, 18 and 24 h irradiation, respectively; the white LED induced larger decreases of 13.2, 19.0 and 25.1% over the same time intervals (Figure 5B).

These findings suggest that photodegradation of TO decreases lipophilic antioxidant activity (DPPH radical scavenging) while enhancing antioxidant response, as measured by ABTS radical scavenging. The ABTS radical scavenging assay operates in both aqueous and organic phases, detecting antioxidants across a wide spectrum of polarity [33]. Conversely, DPPH radical scavenging activity is better expressed by lipophilic compounds using organic solvents such as methanol [34]. The light-induced breakdown of curcuminoids into smaller, more hydrophilic phenolic compounds (Figure 4D) likely explains the decrease in DPPH radical scavenging activity and the concurrent increase in ABTS radical scavenging activity. This behavior aligns with earlier reports showing that photodegradation shifts curcuminoid profiles toward hydrophilic degradation products, which alters their radical scavenging characteristics [16].

### 2.4. Photosensitizing Property of TO

**Figure 6:**
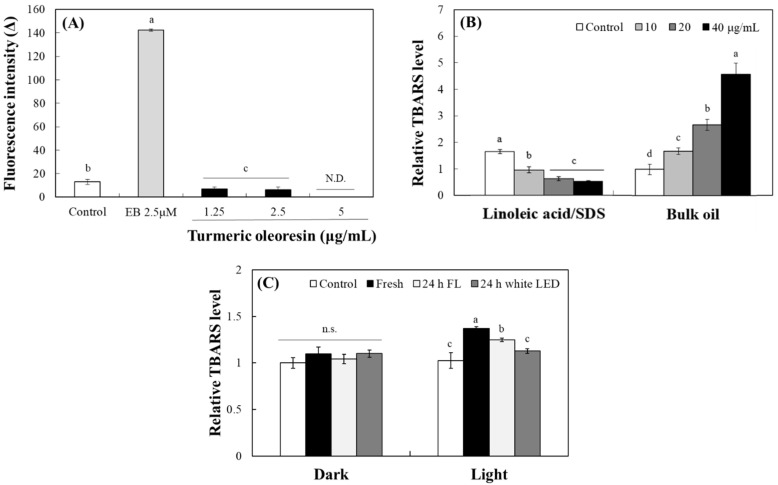
Effects of TO on ROS generation and lipid peroxidation under light. DCFH reacted with different concentrations of TO or EB under 10 W/m^2^ fluorescent light over the course of 1 h (**A**). Levels of TBARS formed in linoleic acid/SDS (0.2/0.4%) emulsion or a bulk oil system containing 10–40 μg/mL TO (vehicle control DMSO 1%) were analyzed after 3 h irradiation of 10 W/m^2^ with a white LED (**B**). TO was also stored under irradiation with fluorescent light (FL) and a white LED for 24 h. Levels of TBARS in a bulk oil system containing fresh or irradiated TO (each 10 μg/mL, vehicle control DMSO 1%) were analyzed after 3 h irradiation with a white LED (10 W/m^2^) or in the dark (**C**). Each value represents the mean ± SD (*n* = 3). Different letters indicate a significant difference (*p* < 0.05) based on a one-way ANOVA with Tukey’s HSD test. N.D.—not detected. n.s.—not significant.

The photosensitizing property of turmeric pigment has been well established, with numerous studies highlighting curcumin’s potential for photodynamic therapy in microbial control, inflammation, and cancer treatment [35,36]. The typical pattern of the actions of photosensitizers generates reactive oxygen species (ROS) under light exposure. To analyze the levels of ROS generated from photosensitizers, a 2′,7′-dichlorofluorescein (DCFH) probe was applied [37]. Upon deacetylation of DCFH-diacetate, DCFH reacted with ROS to yield fluorescent DCF. After 1 h of exposure to fluorescent light, erythrosine B (EB), a positive control, produced a robust increase in DCF fluorescence (Figure 6A). In contrast, incubation of DCFH with TO under identical irradiation conditions rather suppressed DCF fluorescence at concentrations from 1.25 to 5 µg/mL. These results indicate that TO does not generate measurable ROS or promote DCFH oxidation under these conditions, suggesting that ROS may not be produced directly by the photosensitizing actions of curcuminoids (Figure 6A).

Lipids are crucial components of living organisms and are highly susceptible to oxidation by photosensitizers under light. Malondialdehyde, an oxidative decomposition product produced during lipid peroxidation, reacts with thiobarbituric acid (TBA) to produce TBA-reactive substances (TBARSs) [38]. Accordingly, the photosensitizing property of TO was evaluated by measuring its ability to induce lipid peroxidation under light conditions, as determined by TBARS formation. In an oil-in-water (O/W) emulsion system containing linoleic acid/sodium dodecyl sulfate (SDS), TO rather concentration-dependently inhibited TBARS production under light, reflecting its residual antioxidant activity (Figure 6B). Conversely, in a bulk canola oil system, TO enhanced lipid peroxidation in a concentration-dependent manner, confirming its pro-oxidant and photosensitizing action. The results indicate that the photosensitizing property of curcuminoid cannot be adequately assessed in an aqueous environment due to its hydrophobic nature. Instead, the photosensitivity of TO is better expressed in hydrophobic environments, where curcuminoids readily partition into the nonpolar phase and directly interact with a lipid substrate.

To assess the effect of prior photodegradation on this activity, TO samples were pre-irradiated for 24 h under fluorescent light or white LED (10 W/m^2^) or stored in the dark. Then, the samples were incubated with canola oil under the white LED for 3 h (Figure 6C). Fresh TO significantly induced lipid peroxidation, as evidenced by TBARS formation upon irradiation; pre-irradiated TO induced progressively less peroxidation. Notably, TO degraded under fluorescent light significantly reduced TBARS formation, and TO pre-irradiated with white LED returned TBARS levels to baseline (Figure 6C). These results strongly suggest that curcuminoids in TO lose their intrinsic activity, such as photosensitizing properties, due to photodegradation when exposed to light.

### 2.5. Measurement of the Photosensitivity of TO Using an MTT Formazan Probe

**Figure 7:**
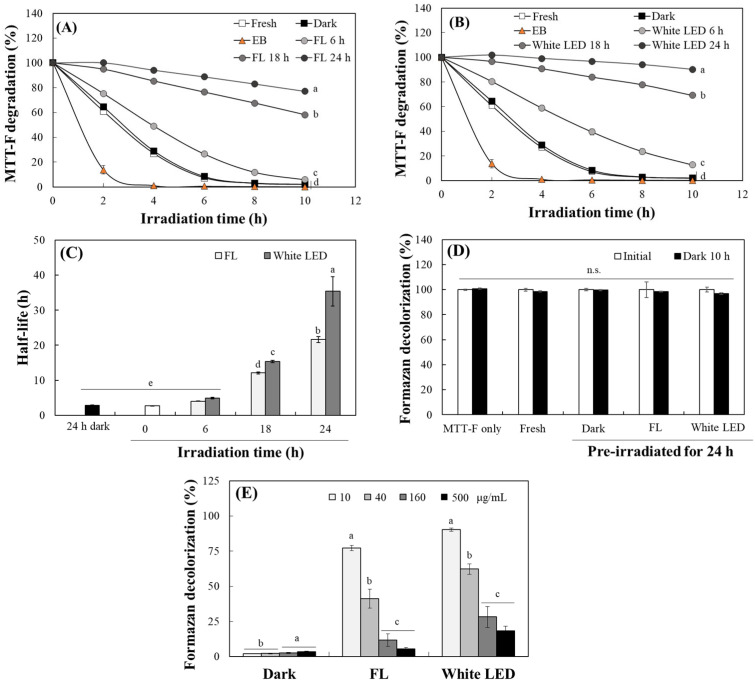
Analysis of the photosensitizing property of TO and its photodegradation products using the MTT-F probe. TO (10 μg/mL) was irradiated under fluorescent light (FL) and white LED light for 6, 18, and 24 h or 24 h in the dark. EB (2.5 μM), fresh turmeric, and the photodegradation products (each 10 μg/mL) under FL (**A**) or white LED (**B**) were incubated with 200 μM MTT-F under white LED (10 W/m^2^), and decolorization of MTT-F was measured at each time point over the course of 10 h. The half-lives for formazan decolorization were calculated (**C**). Residual formazan levels in the presence of 24 h pre-irradiated TO (10 μg/mL) after 10 h incubation in the dark (**D**). Residual color intensity of MTT-F with TO (10 μg/mL) pre-treated under lights or in the dark for 24 h at different concentrations are also shown (**E**). Each value represents the mean ± SD (*n* = 3). Different letters indicate significant difference (*p* < 0.05) based on a one-way ANOVA with Tukey’s HSD test. n.s.—not significant.

The photosensitizing activity of TO was further evaluated by monitoring the decolorization of 1-(4,5-dimethyl thiazol-2-yl)-3,5-diphenylformazan (MTT-F) [37]. This method allows for direct measurement of photosensitizing activity, as the photosensitizer induces the oxidation and bleaching of formazan under light irradiation. MTT-F dissolved in DMSO was exposed to 10 W/m^2^ white LED light in the presence of TO (10 µg/mL) or EB (2.5 μM). EB, used as a positive control, elicited rapid and irradiation time-dependent formazan decolorization, confirming the responsiveness of the assay. Significant decolorization of MTT-F by TO also occurred with increasing irradiation time under white LED exposure over the course of 10 h (Figure 7A,B). Notably, both fresh TO and TO stored in the dark for 24 h bleached over 90% of the MTT-F within 6 h. However, pre-irradiation of TO under fluorescent light (Figure 7A) or white LED (Figure 7B) for 6, 18 or 24 h progressively attenuated to decolorize MTT-F. Quantitatively, the time required to achieve 50% decolorization (half-life) significantly increased from ~3 h to 21.6 h for dark-stored TO and to 35.4 h for TO pre-irradiated for 24 h under fluorescent light and white LED light, respectively (Figure 7C). No significant decolorization, however, occurred in the dark control (Figure 7D).

Additionally, it was observed that higher concentrations of TO during pre-light irradiation resulted in less formazan decolorization (Figure 7E). This suggests that less decomposition occurs at higher concentrations under light, which is consistent with the results presented in Figure 2 and Figure 3 regarding the photostability of TO. These findings collectively indicate that intact curcuminoid chromophores act as efficient photosensitizers, likely via Type I electron-transfer processes that oxidize MTT-F. Conversely, photodegradation of the chromophore leads to a marked decrease in this activity. The extent of curcuminoid degradation directly affects the residual photosensitizing potential; higher levels of turmeric pigment retain more intact chromophores and, consequently, induce the decolorization of formazan more effectively.

### 2.6. Changes in Cytotoxic Activity by Photodegradation of TO

**Figure 8:**
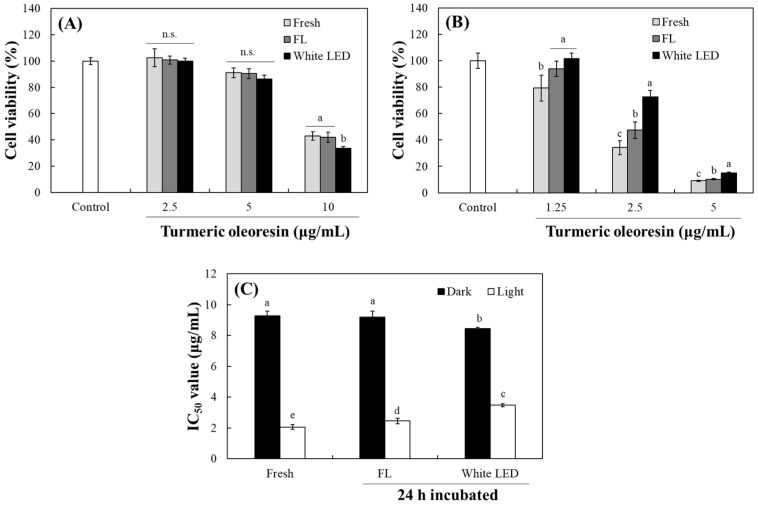
Cytotoxic effects of TO and its photodegradation products on B16F10 melanoma. TO was irradiated under fluorescent light (FL) and white LED for 24 h. B16F10 cells were treated with different concentrations (1.25–10 μg/mL) of fresh or the pre-irradiated TO. The cells were then incubated in the dark (**A**) or after irradiation of white LED (5 W/m^2^) for 30 min (**B**) and further incubated for 24 h in the dark at 37 °C, with 5% CO_2_. The cell viability was analyzed by MTT assay. IC_50_ values were also calculated (**C**). Each value represents the mean ± SD (*n* = 6). Different letters indicate a significant difference (*p* < 0.05) based on a one-way ANOVA with Tukey’s HSD test. n.s.—not significant.

The photosensitizing properties of curcumin and TO against melanoma cells, significantly enhancing their anticancer efficacy with light exposure, have been previously documented [24,39,40]. To investigate how photodegradation alters the cytotoxic profile of TO, B16F10 murine melanoma cells were treated with fresh TO and TO pre-irradiated under fluorescent light or white LED for 24 h. These experiments were conducted by comparing the effects only in the dark and after a 30 min light challenge with white LED before incubation in the dark. In the absence of 30 min irradiation (under continuous dark conditions), photodegraded TO under white LED exhibited slightly higher cytotoxicity than either fresh TO or TO degraded under fluorescent light (Figure 8A). This suggests that photodegradation products generated by white LED might possess intrinsic and light-independent cytotoxic activity, perhaps through enhanced cellular uptake or interaction with distinct biochemical targets.

Upon 30 min of white LED exposure in the presence of cells, all TO formulations demonstrated significantly more potent cytotoxicity (Figure 8B). Fresh TO showed the strongest phototoxic response with an IC_50_ of 1.87 µg/mL. In contrast, TO pre-irradiated under fluorescent light or white LED showed significantly attenuated phototoxicity with IC_50_ values of 2.45 and 3.48 µg/mL, respectively (Figure 8C). Notably, TO subjected to 24 h of white LED photodegradation was the least effective photosensitizer, reinforcing that intact curcuminoid chromophores are the primary mediators of light-activated cell death. These trends of decreased phototoxicity align with our earlier observations of altered lipid peroxidation (Figure 6C) and reduced MTT-F bleaching (Figure 7) following TO photodegradation. Taken together, our results indicate that while certain breakdown products can exert baseline cytotoxic effects in the dark, the robust photodynamic therapeutic potential of TO critically depends on the preservation of its native curcuminoid structure.

Our findings have important practical implications. In contexts where turmeric pigment is exposed to visible light, such as in food, cosmetic, or biomedical applications, its photosensitizing property could inadvertently trigger unwanted oxidative chemistry. This could potentially accelerate product spoilage or induce phototoxicity in skin. Conversely, the deliberate preservation of intact curcuminoids might be strategically exploited in photodynamic antimicrobial or anticancer therapies. Moreover, product formulations should be designed with special consideration of white LED exposure. Future investigations could benefit from examining the relationship between irradiance and degradation across different wavelength bands.

## 3. Materials and Methods

### 3.1. Chemicals and Cell Lines

TO containing 40.3% curcuminoids (61.0%, 21.2%, and 17.8% *w*/*w* for curcumin, DMC, and BMC, respectively) was purchased from ES ingredients (Gunpo, Republic of Korea). Trichloroacetic acid and TBA were obtained from Samchun Chemical Co. (Seoul, Republic of Korea) and VWR International (Poole, UK), respectively. Canola oil was purchased from a local market (Seoul, Republic of Korea). HPLC-grade tetrahydrofuran (THF) was acquired from J.T. Baker Co. (Phillipsburg, NJ, USA). All other chemicals, including MTT-F, were purchased from Sigma Aldrich Chemical Co. (St. Louis, MO, USA). B16F10 murine melanoma cells were obtained from the American Type Culture Collection (ATCC, Manassas, VA, USA). B16F10 cells were cultured in Dulbecco’s modified Eagle’s medium (DMEM), supplemented with 10% fetal bovine serum, 100 unit/mL penicillin, and 0.1 mg/mL streptomycin. The cells were maintained at 37 °C in 95% humidity and 5% CO_2_.

### 3.2. Light Sources and Irradiation

Two distinct light sources were utilized in this study: fluorescent light (27 W, FPL27EX-D, Aris Co., Seoul, Republic of Korea) and white LED (Bissol LED Inc., Seoul, Republic of Korea). The emission wavelength spectra of these lights are presented in Figure 1D and Figure 1E, respectively. Light irradiation intensity was adjusted by varying the irradiation distance for fluorescent light or using the regulator equipped in the LED irradiation device, using a portable solar power meter (TM-207, Tenmars Electronics Co., Taipei, Taiwan). The correlation between the irradiation distance and light intensity was established based on our previous research [37]. The intensity of irradiation was expressed as W/m^2^ (light intensity per unit area).

### 3.3. Analysis of TO Photostability Based on Changes in Color and Fluorescence

TO (100 mg/mL) was aliquoted in DMSO and stored at −80 °C. It was then diluted to concentrations ranging from 20 to 1000 μg/mL, and 200 µL of each dilution was dispensed into a 96-well plate. The plate was irradiated under fluorescent light or white LED (each 10 W/m^2^) at 25 °C for 24 h. The irradiated samples collected at each time point were uniformly diluted to 20 μg/mL with DMSO, and the color intensity was measured at 435 nm. Changes in fluorescence intensity of TO were also analyzed at excitation 440 nm and emission 535 nm (cut-off 530 nm) using a microplate reader (Spectra Max M2, Molecular Device, Sunnyvale, CA, USA).

### 3.4. Analysis of Individual Curcuminoid Levels Using HPLC

Changes in individual curcuminoid levels due to light irradiation were analyzed using HPLC equipped with a diode-array detector (DAD) (Agilent 1100 Series, Agilent Technologies, Waldbronn, Germany) following a previously established method [16]. The mobile phase consisted of a mixture of 40% THF and 60% deionized water containing 1% citric acid (*v*/*v*) adjusted to pH 3 with saturated KOH. The HPLC-packed column C18 (4.6 mm i.d. × 150 mm, 5 μm particle size, Shiseido, Tokyo, Japan) was maintained at 30 °C with a flow rate of 1 mL/min during separation. The injection volume was 20 µL, and peaks were detected at 435 nm. A wavelength of 300 nm was also used to detect colorless degradation products.

### 3.5. Analysis of Antioxidant Activity

Antioxidant properties were measured with reference to previous methods [16]. To evaluate DPPH radical scavenging activity, 100 µL of each sample diluted with DMSO was mixed with 100 µL of 0.375 mM DPPH solution. After 30 min incubation in the dark, the absorbance was measured at 517 nm. A solution of 10 mM ABTS and 10 mM potassium persulfate in a ratio of 7.4:2.6 (*v*/*v*) was stored in the dark at room temperature for 24 h to prepare an ABTS radical. This solution was then used after 33-fold dilution with distilled water. Subsequently, 150 µL of the diluted ABTS radical solution was added to 50 µL of each sample (containing 4% DMSO). After 30 min, the scavenging activity was evaluated at 734 nm using a microplate reader (Spectra Max M2).

### 3.6. Effects of TO on ROS and Lipid Peroxidation

ROS generation from TO under light was analyzed using a DCFH fluorescence probe [37]. DCFH was generated by incubating 25 µM DCFH-diacetate with HCT 116 cells (80–90% confluency in a in 100 φ dish) at 37 °C for 2 h. The cells were lysed with 1 mL DMSO per dish. The cell lysate containing DCFH was diluted 10-fold with distilled water and mixed with TO or EB diluted in distilled water. The reaction mixture was incubated for 1 h in the dark or under fluorescent light (10 W/m^2^), and changes in DCF fluorescence were analyzed at emission 535 nm and excitation 485 nm (cut-off 530 nm) using a microplate reader. Induction of lipid peroxidation by TO under light was evaluated based on a previously described method [24]. Different concentrations of TO were directly added to canola oil using 1% DMSO as a vehicle, and the mixture was exposed to white LED (10 W/m^2^). After 3 h irradiation, 180 µL of each collected sample was mixed with 90 µL of trichloroacetic acid (1 M), 90 µL of 0.8% TBA, and 15 µL of 5% butylated hydroxytoluene. The mixture was vigorously stirred for 10 min using a mixing block (MB-102, Bioer Technology, Hangzhou, China). It was then boiled at 97–100 °C for 7 min, left on ice for 4 min, and then centrifuged at 10,000× *g* for 3 min (Mini, Gyrozen Co., Gimpo, Republic of Korea). Formation of TBARS in a supernatant solution was measured at 532 nm. For the preparation of an O/W system, equal volumes of TO in 0.4% SDS and 0.2% linoleic acid in 0.4% SDS were mixed. The mixture was exposed to light under the same conditions as above, and TBARS levels were measured in the same manner.

### 3.7. Measurement of Photosensitizing Activities of TO Using MTT Formazan Probe

The photosensitizing activities of fresh or pre-irradiated TO were analyzed using MTT-F as a probe [37]. Pre-irradiated TO was prepared after different periods of irradiation under fluorescent light or white LED (each 10 W/m^2^). The fresh or pre-irradiated TO for 6, 18, and 24 h in DMSO (50 μL) was mixed with 50 μL of 400 μM MTT-F solution. The reaction mixture was exposed under white LED (10 W/m^2^), and the formazan decolorization was measured at 500 nm for 10 h.

### 3.8. Determination of Cytotoxic Effects

Changes in cytotoxicity according to the photodegradation of TO were evaluated using the 3-(4,5-dimethylthiazol-2-yl)-2,5-diphenyltetrazolium bromide (MTT) assay. B16F10 melanoma cells were seeded at 0.5 × 10^4^ cells per well of a 96-well plate and grown for 24 h. Cells were then treated with different concentrations (1.25–5 or 2.5–10 μg/mL) of TO (fresh or pre-irradiated for 24 h under fluorescent light or white LED light (10 W/m^2^)) in serum-free and phenol red-free media containing 1% DMSO as a vehicle. Another experimental group of cells was subsequently exposed to white LED (5 W/m^2^) for 30 min under the lid of a standard 96-well culture plate in a CO_2_ incubator, with all other light sources blocked. After 24 h incubation in the dark, the treated medium was removed, and 100 μL of 0.5 mg/mL MTT diluted in serum-free medium was added to each well. After 45 min incubation at 37 °C, the medium was replaced with 100 μL of DMSO in each well, and absorbance was measured at 550 nm.

### 3.9. Data Analysis

Each experiment was repeated more than three times, and all values are presented as mean ± standard deviation. Statistical significance was evaluated using Student’s *t*-test. A one-way ANOVA with Tukey’s HSD (honestly significant difference) test was used to compare multiple results using the SPSS program (IBM SPSS Statistics 24, SPSS Inc., Chicago, IL, USA).

## 4. Conclusions

This study demonstrated that turmeric pigment is highly susceptible to photodegradation from visible light, with irradiation from a white LED accelerating the loss of absorbance and fluorescence relative to fluorescent light. HPLC analysis confirmed that curcumin, DMC, and BMC undergo progressive decomposition under both light sources, accompanied by the emergence of smaller hydrophilic degradation products. Photodegradation alters the antioxidant profile of TO. Scavenging activity against DPPH radical declined, whereas the ABTS radical scavenging activity was enhanced. Furthermore, the photosensitizing properties of turmeric pigment diminished in parallel with the breakdown of curcuminoids. Intact TO efficiently induced lipid peroxidation in a bulk oil system and decolorization of MTT-F. However, pre-irradiated TO under both light conditions showed delayed formazan decolorization and reduced lipid peroxidation. In B16F10 melanoma cells, phototoxic efficacy decreased in parallel with curcuminoid loss, although certain photoproducts retained their cytotoxicity in the dark. Collectively, these findings underscore that the functional lifespan of turmeric pigment under light depends on the intricate interplay between structural integrity and degradation chemistry. To preserve both color and bioactivity for food and cosmetic applications, it is crucial to minimize blue-rich light exposure or employ photoprotective formulations. Conversely, it is essential to maintain the integrity of curcuminoids for photodynamic therapy, which requires the development of strategies to prevent their premature photodegradation.

## Data Availability

The original contributions presented in this study are included in the article. Further inquiries can be directed to the corresponding author.
